# Modified Ilioinguinal Approach to Treat Pelvic or Acetabular Fractures: A Retrospective Study

**DOI:** 10.1097/MD.0000000000001491

**Published:** 2015-09-18

**Authors:** Yongliang Yang, Qinghu Li, Haomin Cui, Zhenhai Hao, Yonghui Wang, Jian’an Liu, Lianxin Li, Dongsheng Zhou

**Affiliations:** From the Department of Orthopedic Trauma, Shandong Provincial Orthopedic Hospital, Shandong Provincial Hospital affiliated to Shandong University, Jinan, China.

## Abstract

The aim of this study was to evaluate the potential advantages and clinical results of a modified minimally invasive ilioinguinal approach for the treatment of acetabular or pelvic fractures to the results obtained using a standard ilioinguinal approach.

Forty-six patients who were diagnosed as having anterior column acetabular fractures or anterior pelvic ring fractures underwent open reduction and internal fixation through 2 different surgical approaches between June 2008 to June 2012 in our trauma center was studied. The modified ilioinguinal group included 20 patients and the other 26 patients were in the standard ilioinguinal approach group. The clinical and radiographic results were recorded and compared between the 2 groups.

There were no significant differences between 2 groups in the mean age, sex, fractures type, and causes of acetabular or pelvic fractures. The mean blood loss in the modified group was 560.0 ± 57.3 mL versus 850.0 ± 59.0 mL in the standard ilioinguinal group. The operative time was significantly reduced with modified ilioinguinal approach (86.0 ± 4.56 min vs. 101.9 ± 5.38 min). The mean hospital stay was 16.8 ± 0.58 days and 18.7 ± 0.52 days in the modified and standard ilioinguinal groups, respectively. According to the Matta score, the quality of reduction between the 2 groups was not significantly different. The complication rate was low in the modified group but not significantly different between the 2 groups. Forty-two patients were followed up with clinical examination and radiographs at a mean of 15.2 months. Solid union was observed in 42 cases at a mean time of 14.8 weeks. The mean Harris Hip Score and the Majeed scores at the time of evaluation were not significantly different between the 2 groups.

On comparing the 2 surgical ilioinguinal approaches, it was found that using modified ilioinguinal approach decreased operative time and blood loss, and did not affect the quality of fracture reduction and fracture healing. This study demonstrates that the modified ilioinguinal approach is a simple and minimally invasive approach for anterior column acetabular fractures and pubic rami fractures comparing with the standard ilioinguinal approach.

## INTRODUCTION

Surgical management of displaced acetabular or pelvic fractures is widely accepted in most trauma units. The surgical decision making entails classification of the fractures and operative approach. The choice of operative approach is dependent on the fracture type, direction of displacement, skin situation at the surgical incision site, and duration from initial injury.^1–4^ Generally, the operative approaches to the acetabular fractures can be classified into anterior, posterior, extensile, and combined approaches.^5^ The standard anterior approach for treatment of acetabular fractures is the ilioinguinal approach described by Letournel in 1961.^1^ This approach exposes the anterior wall, respects the anatomical structures of the pelvis, and allows broad visual and tactile exposure to the entire anterior ilium, from the anterior column to the sacroiliac joint, the linea terminalis and the inner aspect of the posterior column.^6–9^ However, the fact that the entire anterior part of the abdominal wall has to be detached from the ilium or the inguinal ligament in order to open the “middle window” may result in soft tissue complications. The main complications comprise high rates of postoperative wound infections and iatrogenic injury to the femoral nerve and the iliofemoral blood vessels.^6,10–13^

This study presents our experience of internal fixation of acetabular fractures and pelvic fractures using a modified, minimally invasive ilioinguinal approach, which provides closer visualization to the entire anterior column, the pelvic brim portion of the posterior column, and permits stable internal fixation of the acetabular fracture with mainly anterior displacement and the rami and iliac fracture components of the pelvic fracture through the sneak path under the femoral vessels. The aim of this study was to evaluate the safety, efficacy, and benefits of the modified versus the standard ilioinguinal approach for the treatment of the acetabular anterior column or the anterior and lateral parts of pelvic fractures.

## METHODS

**FIGURE 1:**
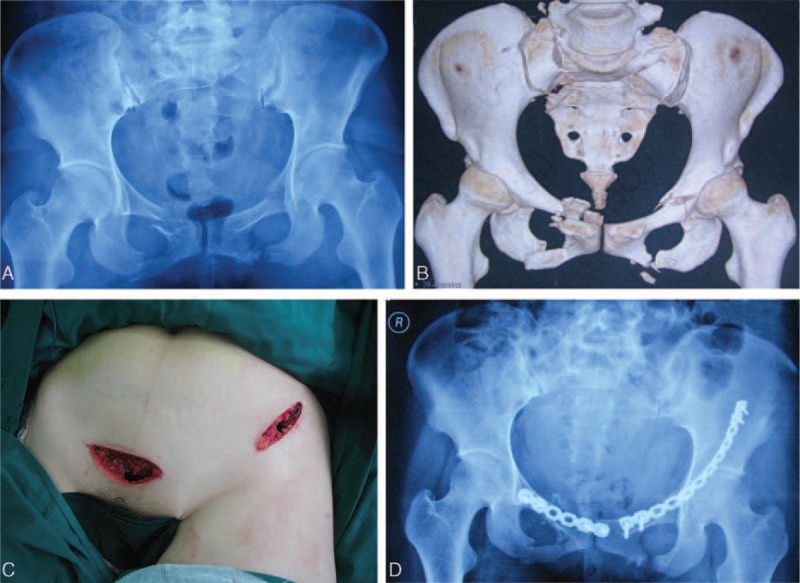
A 28-year-old man suffered from pelvic fractures. A, The anteroposterior radiograph shows the fractures of bilateral pubic rami and sacroiliac joint of the pelvis (Type 61-B1, according to AO/OTA classification). B, Three-dimensional CT reconstruction image shows the fracture clearly and open-book dislocation of sacroiliac joint. C, It shows the minimally invasive ilioinguinal approach, which was composed of the lateral and the medial portions of the standard ilioinguinal approach. D, Postoperative anteroposterior radiograph after the internal fixation of the pelvic fracture shows the good quality of fracture reduction.

**FIGURE 2:**
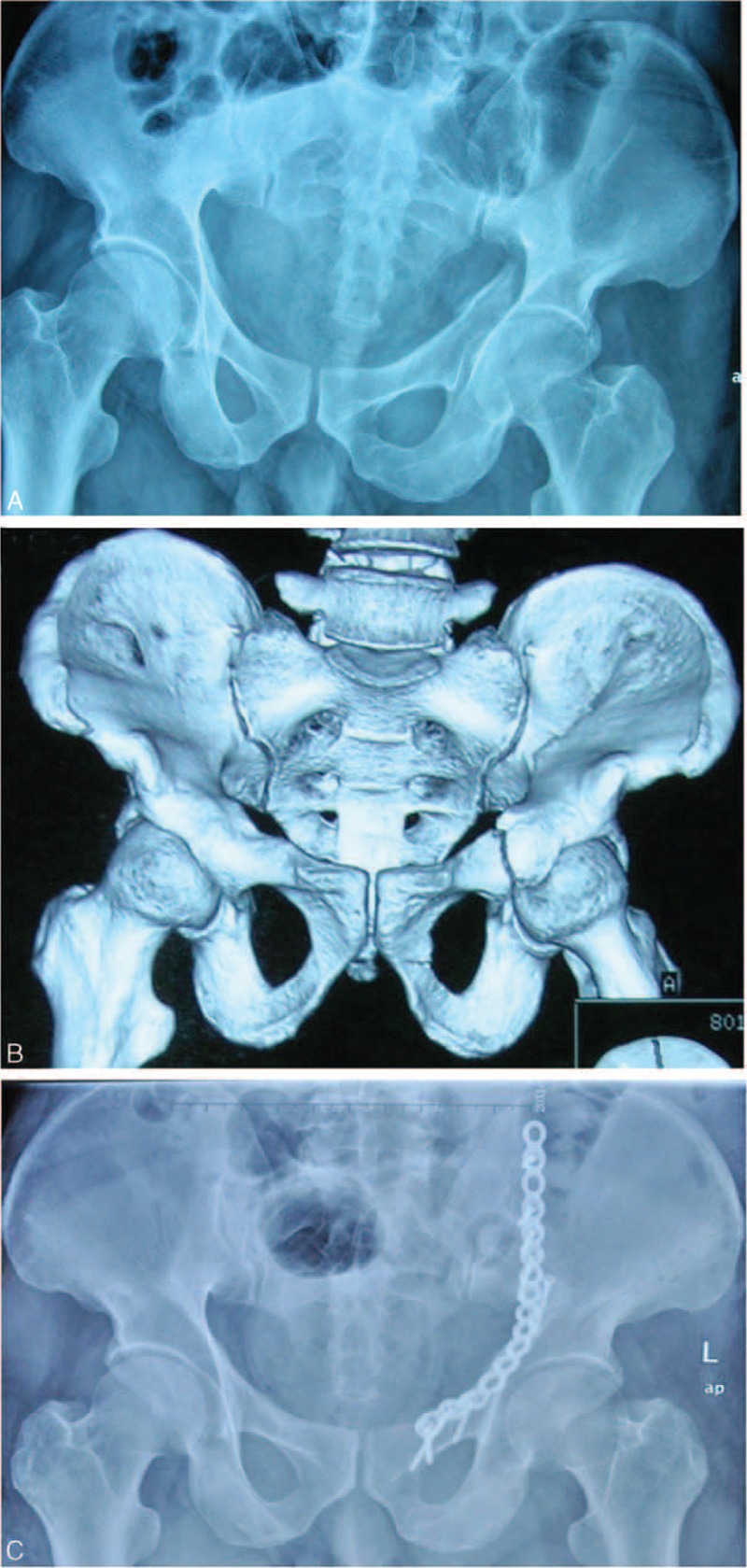
A 51-year-old man suffered from acetabular fracture. A, The anteroposterior radiograph shows the acetabular anterior column fractures (Type 62-A3, according to AO/OTA classification). B, Three-dimensional CT reconstruction image shows the acetabular fracture clearly, and a fracture line across the iliac bone. C, Postoperative anteroposterior radiograph after the internal fixation of the acetabular fracture shows a good reduction of the acetabular fossa and the ilium.

From June 2008 to June 2013, a total of 644 patients who had suffered from pelvic or acetabular fractures were treated in Shandong Provincial Hospital affiliated to Shandong University. Thirty-one patients diagnosed as acetabular anterior column fractures (Type 62-A3, according to AO/OTA classification system) and 15 patients diagnosed as fractures of the rami and iliac crest or sacroiliac joint of the pelvic fractures (Type 61-B1 and B2, according to AO/OTA classification system) underwent open reduction and internal fixation through the anterior approaches (Figures 1 and 2). This study was approved by the institutional review board of Shandong Provincial Hospital affiliated to Shandong University. Twenty patients were operated through the modified minimally invasive ilioinguinal approach. During the same time period, the other 26 patients underwent surgeries by the standard ilioinguinal approach. All procedures were carried out by 2 experienced surgeons. Thirty-two cases were male and 13 were female. The average age was 41.1 ± 2.9 years in the modified group and 43.2 ± 2.47 years in the standard group. The causes of acetabular or pelvic fractures included traffic accidents, fall injuries, and crush injuries. Associated injuries: 13 cases with limb fractures, 5 cases with chest or abdominal injuries, and 4 cases with head injuries. The time from trauma to surgery was 7.5 ± 1.22 days. In total, 21 patients were transferred from other local hospitals.

The pelvic radiographs, including anteroposterior, computed tomography (CT) scans, and three-dimensional reconstruction, were applied in all cases. Iliac and obturator oblique views of radiographs were applied for acetabular fractures. Inlet and outlet views of radiographs were applied for pelvic fractures. All patients underwent femoral condyle or tibia tubercle skeletal traction after admission (Figure 1A, B).

Data on the clinical outcome, operative time, operation-related complications, blood transfusion, quality of reduction, and length of hospital stay were collected and reviewed. Operative time was obtained from the anesthesia record and defined as the time from the first incision to closure of the incision. The quality of fracture reduction was assessed by postoperative x-rays and CT scan, and then classified according to Matta reduction criterion. Criteria were: excellent 4 mm or less, good 5 to 10 mm, fair 10 to 20 mm, and poor more than 20 mm of displacement.^14^ One experienced orthopedic surgeon and one radiographer, not involved in the treatment team, were asked to review all films and assess the quality of reduction. If the 2 observers had controversies in postoperative radiographs, a third high-level orthopedic surgeon was invited to assess radiographs and make a decision.

After general endotracheal intubation anesthesia was induced, the patient was positioned supine or floating lateral on a radiolucent operating table, with the ipsilateral extremity draped free. The fracture was identified under fluoroscopy and the incisions were marked. The standard ilioinguinal approach as described by Letournel was used in 26 cases.^6–9^ The acetabular or pelvic fracture was exposed through 3 surgical windows. First window situated between the iliopsoas and the lilac crest, second window between the inguinal vessels and the iliopsoas, third window between the spermatic cord and the inguinal vessels being careful to avoid of damaging in the operation.

**FIGURE 3:**
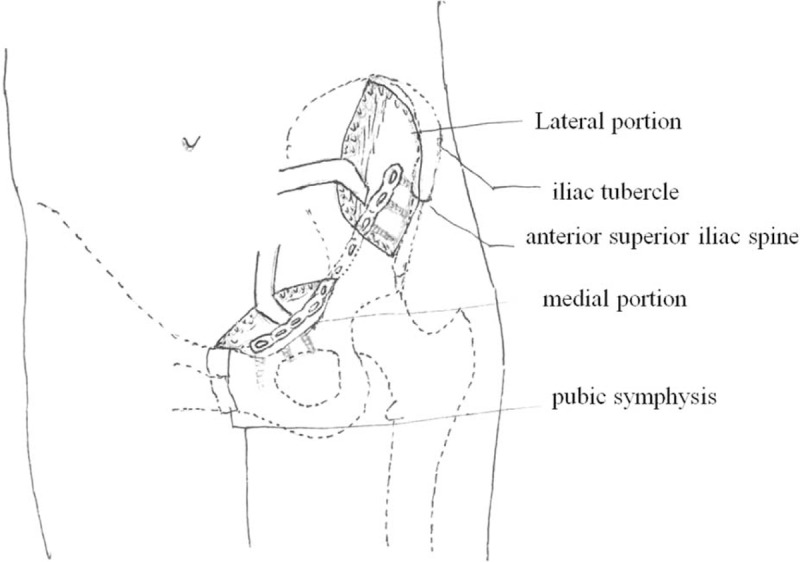
The diagrams of the modified ilioinguinal approach.

**FIGURE 4:**
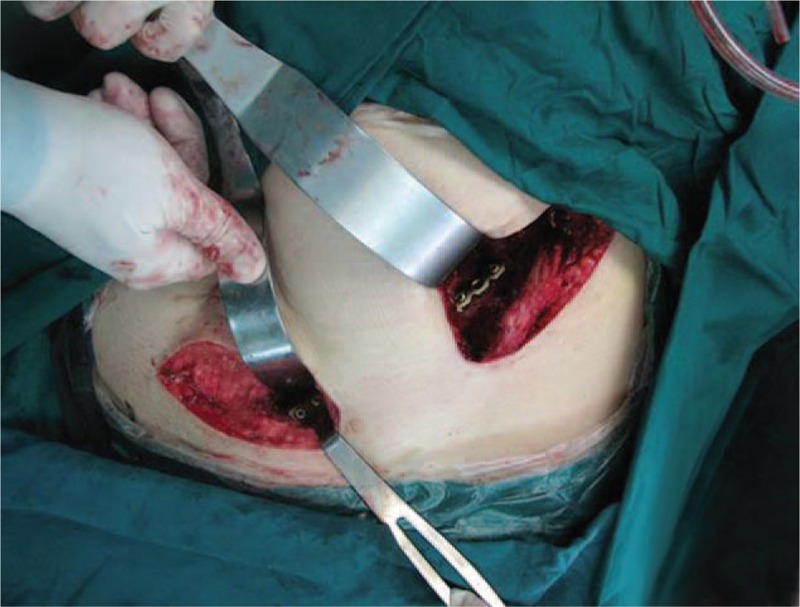
The 2 windows of the approach and the plate placed beneath the deep fascia.

The minimally invasive ilioinguinal approach was composed of the lateral and the medial portions of the standard ilioinguinal approach (Fig. 1C). A Pfannenstiel approach could be used instead of medial portion of ilioinguinal approach. The primary transverse incision was about 1 to 2 cm above the pubic symphysis, extending outward from the ipsilateral pubic tubercle; the length was about 4 to 6 cm. The spermatic cord or round ligament must be separated and protected. The insertions of inguinal ligament, pectineal ligament, and pectineus muscle were incised. Extraperitoneal dissection was done and the pubic symphysis was exposed. The lateral incision started at the junction of the posterior and middle third of the iliac crest, passing the anterior superior iliac spine and curving toward the pubic symphysis, which was about 3–5 cm in length. The lateral femoral cutaneous nerve must be protected, which passed through or immediately deep to the inguinal ligament, usually adjacent to the anterior superior iliac spine, but could be found at variable locations. At the level of the iliac crest, the abdominal muscles must be detached from the iliac crest without damaging the muscles themselves. The iliac muscle was then freed from the inner side of the iliac crest. The freeing was continued up to the sacroiliac joint and the border of the true pelvis. The 2 parts of the approach were then connected using blunt preparation. At the same time, the knee and hip of the affected side should be flexed for relaxation of the iliopsoas muscle. The preparation followed beneath the deep fascia, using mainly the fingers and protecting the inguinal vessels and nerves against laceration when applying the plate (Figures 3 and 4). Reduction was enabled by the aid of axial traction with mild abduction of the leg performed by the assistant surgeon. The acetabular anterior column fracture was cleaned and indirect reduction was performed by a pusher or a small forceps through the incisions. Thereafter, the fractures were temporarily fixed with K-wires and isolated screws. The sequence of repair followed the usual pattern employed in the standard ilioinguinal approach. After anatomical fracture reduction and fixation by lag screws, a preshaped 3.5 mm reconstruction plate was inserted through the distal incision. At least 3 screws were inserted into pubic rami and ilium to fix the plate through the medial and lateral incisions respectively, avoiding inserting into the hip joint. The positions of plate and screws and reduction of the fractures were checked again with intro-operative C-arm fluoroscopy.

Two drainage tubes were placed into 2 incisions separately and removed 48–72 hours postoperatively. The antibiotic was routinely used until the drainage removed to prevent infection. The continuous passive motion machine was used in the affected leg and each patient was encouraged to do isometric exercises. The sitting position was recovered and hip joint was flexed and extended actively 3–4 days postoperative. Partial weight-bearing with crutches or a walker began 4 weeks postoperative and total weight-bearing was achieved 3 months after operation.

Parametric data, such as operative time, fluoroscopic time, and blood loss, were described as mean ± SEM and compared using Student *t* tests. Proportional values were compared using *χ*^2^-analysis or Fisher exact test where applicable. For each test, a *P* value <0.05 was defined as significant. We calculated that a sample size of 14 patients per group would give 90% power to detect a difference between groups for the primary endpoint (=0.05, 2-tailed). So the case numbers in our study are reasonable to assure valid result.

## RESULTS

Patient Characteristics. Of 46 cases included in this study, 26 cases were operated through the standard ilioinguinal approach, whereas the other 20 cases were by the modified minimally invasive ilioinguinal approach. The patients’ demographics are summarized in Table 1. There were no significant differences in age, sex, type of fractures, and causes of pelvic or acetabular fractures.

**TABLE 1:**
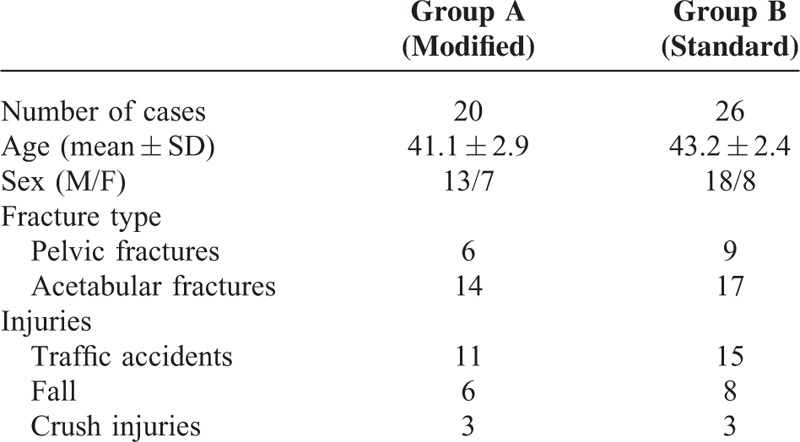
Demographics and Disease Distribution of Patients in This Study

Operative Records. The mean operative time in the modified ilioinguinal approach group was 86.0 ± 4.56 minutes (50–120 min) compared with 101.9 ± 5.38 minutes (60–180 min) in the standard ilioinguinal approach group (*P* = 0.0351). The mean blood loss was 560.0 ± 57.3 mL (200–1000 mL) in the modified ilioinguinal group and 850.0 ± 59.0 mL (300–1500 mL) in the standard ilioinguinal group (*P* = 0.001). The mean hospital stay was 16.8 ± 0.58 days in the modified ilioinguinal group and 18.7 ± 0.52 days in the standard ilioinguinal group (*P* = 0.017) (Table 2).

**TABLE 2:**
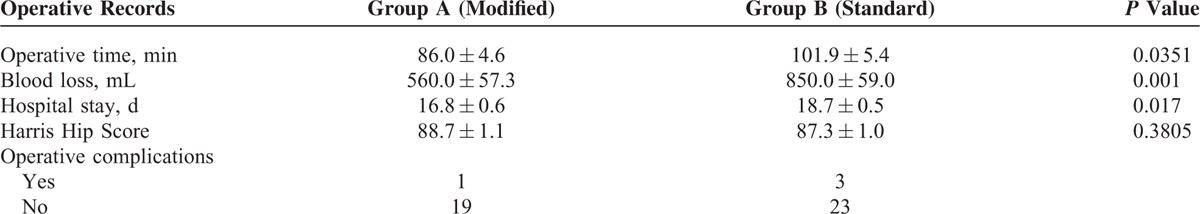
Operative Records of Patients in 2 Groups

Radiographic Assessment. In this study, the postoperative plain radiographs and CT scans were used to assess the quality of reduction according to the methods described by Matta (Figures 1D and 2C). In the modified ilioinguinal group, 12 patients had anatomic results, 7 patients had satisfactory results, and 1 patient had a poor result. Meanwhile, anatomic results were achieved in 15 cases, satisfactory results in 9 cases, and poor results in 2 cases in the standard ilioinguinal group, respectively. The quality of reduction between the 2 groups was not significantly different (*P* > 0.05).

Complications. No procedure-related deaths were identified in all cases. In the modified ilioinguinal group, fat liquefaction was found in 1 case, and the wound healed after dressings change. In the standard ilioinguinal group, 1 patient complained of lateral femoral skin hypoesthesia after surgery, maybe related to lateral femoral cutaneous nerve injury, but his symptom disappeared 1 month postoperatively. The corona mortis was injured in 1 patient resulting in bleeding during operation. This required the surgeon to tie off the injured vessel to stop the bleeding and transfuse 4 unit red blood cells and 400 mL blood plasma immediately. The patient's vital signs were stable and no sign of bleeding was found after surgery. One case of superficial infection was found in the standard ilioinguinal group and the wound healed after debridement and sensitive antibiotics treatment. No signs of femoral nerve or vascular injuries, venous thrombosis, inguinal hernia, heterotopic ossification, bone nonunion, osteoarthritis, and avascular femoral head necrosis were found in these 2 groups.

Clinical Outcome. Forty-two patients were followed up with clinical examination and radiographs at 1, 3, 6, and 12 months after surgery and then every 6 months. Four patients were lost to follow-up, and 2 patients in each group. The mean follow-up time was 15.2 months (6–36 months). Solid union was observed in 42 cases. The mean time of solid union was 14.8 weeks (12–24 weeks) based on postoperative plain radiographs or CT scans. The mean Harris Hip Score at the time of evaluation was 88.7 ± 1.07 points and 87.3 ± 1.04 points in the modified and standard groups, respectively (*P* = 0.3805). By the Majeed scoring system^15^ at the last follow-up, the good and excellent rate was 94.4% (excellent: 10 cases, good: 7 cases, fair: 1 case) in the modified group versus 91.7% (excellent: 10 cases, good: 12 cases, fair: 2 case) in the standard group.

## DISCUSSION

The modified ilioinguinal approach directly or indirectly exposed the same surgical areas as the classic ilioinguinal approach. This modified approach includes 2 minimal incisions; the lateral incision is from iliac tubercle to anterior superior iliac spine, which is extended according to the type of fractures. The sacroiliac joint, medial portion of ilium, anterior inferior iliac spine, and anterior column of the acetabulum are exposed by the lateral incision. The pubic symphysis, pubic tubercle, iliopubic eminence, and corona mortis are exposed by the medial incision. In order to protect the inguinal vessels and nerves, the lateral and medial incisions must be connected through a subperiosteal channel to facilitate fracture reduction and plate placement. Periosteal stripping needs special techniques, which are from outside to inside and from both sides to the middle gradually. Usually, the middle iliopubic ligament is strong and difficult to dissect and requires the use of a long sharp scissor to release it when necessary. In this study, 20 cases diagnosed as acetabular fractures and pelvic fractures were in the modified group and the other 26 cases were in the standard group. The quality of fracture reduction was not significantly different between the 2 groups on the basis of postoperative radiographs. This indicated that the modified approach was enough to expose the surgical site for fracture reduction and fixation.

Previous studies in the literature revealed that the operative time varied from 175 to 253 minutes due to the different composition of the patient cohorts.^2,10,11^. In this study, the average operative time in the standard ilioinguinal group was 101.9 ± 5.38 minutes (70–180 min), and 86.0 ± 4.56 minutes through the modified ilioinguinal approach. In our opinion, the short average operative time compared with the published data may be related to the simple fracture type. All the cases in this study were diagnosed as anterior column of acetabular fractures and pubic rami fractures, not including complex fractures. However, the average of operative time by the modified ilioinguinal approach was significantly less than that with the standard ilioinguinal approach.

The modified approach not only shortened operative time, but also reduced operative blood loss. Previous studies revealed the blood loss with ilioinguinal approach was from 732 to 1630 mL.^9,12^ In this study, the mean blood loss was 560.0 ± 57.3 mL (200–1000 mL) in the modified ilioinguinal group and 850.0 ± 59.0 (300–1500 mL) in the standard ilioinguinal group. The average blood loss with the modified ilioinguinal approach was less than that by the standard ilioinguinal approach. Previous research revealed that the blood loss in most cases was not caused by the approach but by the bleeding during cleaning and reduction.^16^ In our opinion, the blood loss was less with modified approach as a consequence of the faster operative time and simple fracture type with less soft tissues dissection.

The modified ilioinguinal approach did not affect the quality of fracture reduction in this study. The surgeons must be familiar with the displacements of different types of fractures and the methods of reduction, due to not looking at the fracture site directly. Usually, a Kirschner wire is drilled into the pubic ramus to reduce the fracture from distal to proximal. Sometimes, a poking reduction is needed, which means reduction by poking and using one of the fracture ends as the fulcrum and prizing and poking the other fracture end along the direction necessary for reduction, or fracture is touched and reduced with fingers. If good reduction is achieved, temporary fixation will be achieved with a Kirschner wire, and then a contoured reconstruction plate is placed from the lateral to medial incision through the subperiosteal tunnel. The quality of fracture reduction was assessed by Matta, and there was no significant difference between the 2 groups.

It is better to place the reconstruction or locking plate along the linea terminalis of pelvis to fix the anterior column of acetabular fractures or pubic rami fractures.^17^ But it is difficult to preshape and place the plate due to the pelvic irregular linea terminalis. The plate will need not only anteroposterior prebending, but also lateral and rotary prebending to attach the pelvic contour. There is the risk of screw penetrating into the hip joint or inguinal nerve and vessels injuries.^18^ In this study, the authors changed a little the methods of the plate placement to adapt the minimally invasion ilioinguinal approach. Our previous study demonstrated that the bone quality from pubic tubercle through iliopubic eminence to the iliac was thick and strong; we called it the iliopubic anterior column.^19^ If the plate was placed on the iliopubic anterior column, only anteroposterior prebending plate would be enough. This method of plate placement was suitable for the minimally invasive ilioinguinal approach. In this study, the plate was placed on the iliopubic anterior column in the modified group. The Harris Hip Score and Majeed scoring system between the 2 groups were not different significantly.

The ilioinguinal approach is the classic approach for pubic rami and anterior column of acetabular fractures, but it is more complex to expose inguinal nerve and vessels, resulting in high complication rate, including thromboses, hernias, lymphedema, meralgia paraesthetica, lesions of the femoral vessels, hematoma, and impaired wound healing.^10–13^ As the approach described by Wolf, the modified ilioinguinal approach can reduce the risk of developing some of the complications associated with the standard ilioinguinal approach. Opening of the inguinal canal is not necessary; thus, the integrity of the inguinal floor is not compromised, and the risk of iatrogenic hernia is diminished. Additionally, there is no dissection around the femoral blood or lymphatic vessels.^20^ Furthermore, the corona mortis is often extraperiosteal and located behind the superior pubic ramus at about 33.4 mm (range 21.4–41 mm) away from the symphysis pubis.^21^ So the intact middle window and the subperiosteal channel to reduction and plate placement in the approach can prevent the corona mortis injury. In this study, 1 case suffered fat liquefaction after operation and the wound healed after dressing change. But in the standard ilioinguinal approach, 3 patients suffered different complications, such as lateral cutaneous femoral nerve injury, corona mortis injury, and superficial infection. But no femoral nerve or vessels injuries were found in this study maybe because of less cases in the 2 groups. However, the complication rate was lower in the modified ilioinguinal group, though there was no significant difference between the 2 groups.

Some surgeons reported their results using “Stoppa” or “modified Stoppa” approach as an alternative to the standard ilioinguinal approach. This approach offered additional advantages in comparison to the ilioinguinal approach. The iliopectineal fascia can be incised under direct visualization and the corona mortis can be directly controlled. Meanwhile, it was reported that the complication rate with Stoppa approach was from 18% to 45%, sometimes associated with urinary system diseases.^14,22,23^ Some authors reported that an additional lateral approach was recommended in some patients treated with “Stoppa approach.”^2,23^ The anatomic reduction rate was from 54% to 64% with Stoppa approach in previous literatures.^2,22,23^ In this study, the anatomic reduction rate with modified ilioinguinal approach was higher than Stoppa approach reported in literatures. The modified ilioinguinal approach shortened operative time and reduced soft tissues dissection in comparison with Stoppa approach and the standard ilioinguinal approach.

The indications for the modified ilioinguinal approach include the anterior column of acetabular fractures and pubic rami fractures. If the anterior wall of acetabular fractures was comminuted, the standard ilioinguinal approach instead of modified approach should be applied because the femoral nerve or vessels might be injured during fracture reduction. When it was transverse or both columns of acetabular fracture, the posterior Kocher–Langenbeck (K–L) approach combined with the modified ilioinguinal approach was applied. This modified ilioinguinal approach requires an excellent knowledge of acetabular surgery and the local anatomic structures.

Although significant difference was demonstrated in operative time and blood loss, limitations existed in this study. First, this was a retrospective study, with all the intrinsic shortcomings. Second, due to nonrandom selection, the outcomes may be influenced by the selective bias. Third, the operating surgeons’ experiences were not taken into consideration in the study and that may affect our results. Many surgeons have enough experiences of standard ilioinguinal approach, and may not perceive an additional benefit from the modified ilioinguinal approach. However, in some special acetabular or pelvic ring fractures, the modified invasive ilioinguinal approach, maybe, has a significant advantage.

## CONCLUSIONS

This study yielded an encouraging clinical and radiographic result with modified ilioinguinal approach for treatment of anterior column acetabular fractures and pubic rami fractures. On comparing the 2 ilioinguinal approaches, it was found that the operative time and intra-operative blood loss were reduced though the modified ilioinguinal approach, and did not interfere with the fracture healing and clinical outcomes. This study shows that modified ilioinguinal approach is efficacious and safe for treatment of simple acetabular anterior column or pubic rami fractures.
